# Enterovirus VP1 protein and HLA class I hyperexpression in pancreatic islet cells of organ donors with type 1 diabetes

**DOI:** 10.1007/s00125-025-06384-9

**Published:** 2025-03-17

**Authors:** Teresa Rodriguez-Calvo, Jutta E. Laiho, Maarit Oikarinen, Pouria Akhbari, Christine Flaxman, Thomas Worthington, Paola Apaolaza, John S. Kaddis, Irina Kusmartseva, Sisko Tauriainen, Martha Campbell-Thompson, Mark A. Atkinson, Matthias von Herrath, Heikki Hyöty, Noel G. Morgan, Alberto Pugliese, Sarah J. Richardson, for the nPOD-Virus group

**Affiliations:** 1 Institute of Diabetes Research, Helmholtz Zentrum München, German Research Center for Environmental Health, Munich-Neuherberg, Germany; 2 Faculty of Medicine and Health Technology, Tampere University, Tampere, Finland; 3 Islet Biology Exeter (IBEx), Department of Clinical and Biomedical Sciences, University of Exeter Medical School, Exeter, UK; 4 Department of Diabetes and Cancer Discovery Science, Arthur Riggs Diabetes and Metabolism Research Institute, Beckman Research Institute, City of Hope, Duarte, CA USA; 5 Department of Pathology, Immunology and Laboratory Medicine, College of Medicine, University of Florida, Gainesville, FL USA; 6 Institute of Biomedicine, University of Turku, Turku, Finland; 7 Diabetes Research Institute (DRI), University of Miami Miller School of Medicine, Miami, FL USA; 8 Division of Endocrine, Diabetes and Metabolism, Department of Medicine, University of Miami Miller School of Medicine, Miami, FL USA; 9 Global Chief Medical Office, Novo Nordisk A/S, Søborg, Denmark; 10 Department of Pediatrics, Tampere University Hospital, Tampere, Finland; 11 Fimlab Laboratories, Tampere, Finland; 12 Department of Diabetes Immunology, Arthur Riggs Diabetes & Metabolism Research Institute, Beckman Research Institute, City of Hope, Duarte, CA USA

**Keywords:** Autoimmunity, Enterovirus, HLA-I molecules, Immunofluorescence, Immunohistochemistry, Pancreas, Type 1 diabetes

## Abstract

**Aims/hypothesis:**

Earlier studies of pancreases from donors with type 1 diabetes demonstrated enteroviral capsid protein VP1 in beta cells. In the context of a multidisciplinary approach undertaken by the nPOD-Virus group, we assessed VP1 positivity in pancreas and other tissues (spleen, duodenum and pancreatic lymph nodes) from 188 organ donors, including donors with type 1 diabetes and donors expressing autoantibody risk markers. We also investigated whether VP1 positivity is linked to the hyperexpression of HLA class I (HLA-I) molecules in islet cells.

**Methods:**

Organ donor tissues were collected by the Network for Pancreatic Organ Donors with Diabetes (nPOD) from donors without diabetes (ND, *n*=76), donors expressing a single or multiple diabetes-associated autoantibodies (AAb^+^, *n*=20; AAb^++^, *n*=9) and donors with type 1 diabetes with residual insulin-containing islets (T1D-ICIs, *n*=41) or only insulin-deficient islets (T1D-IDIs, *n*=42). VP1 was assessed using immunohistochemistry (IHC) and HLA-I using IHC and immunofluorescence, in two independent laboratories. We determined assay concordance across laboratories and overall occurrence of positive assays, on a case-by-case basis and between donor groups.

**Results:**

Islet cell VP1 positivity was detected in most T1D-ICI donors (77.5%) vs only 38.2% of ND donors (*p*<0.001). VP1 positivity was associated with HLA-I hyperexpression. Of those donors assessed for HLA-I and VP1, 73.7% had both VP1 immunopositivity and HLA-I hyperexpression (*p*<0.001 vs ND). Moreover, VP1^+^ cells were detected at higher frequency in donors with HLA-I hyperexpression (*p*<0.001 vs normal HLA-I). Among VP1^+^ donors, the proportion with HLA-I hyperexpression was significantly higher in the AAb^++^ and T1D-ICI groups (94.9%, *p*<0.001 vs ND); this was not restricted to individuals with recent-onset diabetes. Critically, for all donor groups combined, HLA-I hyperexpression occurred more frequently in VP1^+^ compared with VP1^−^ donors (45.8% vs 16%, *p*<0.001).

**Conclusions/interpretation:**

We report the most extensive analysis to date of VP1 and HLA-I in pancreases from donors with preclinical and diagnosed type 1 diabetes. We find an association of VP1 with residual beta cells after diagnosis and demonstrate VP1 positivity during the autoantibody-positive preclinical stage. For the first time, we show that VP1 positivity and HLA-I hyperexpression in islet cells are both present during the preclinical stage. While the study of tissues does not allow us to demonstrate causality, our data support the hypothesis that enterovirus infections may occur throughout the natural history of type 1 diabetes and may be one of multiple mechanisms driving islet cell HLA-I hyperexpression.

**Graphical Abstract:**

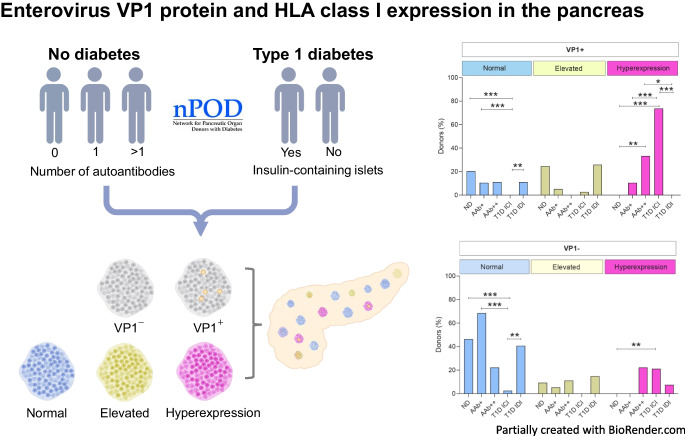

**Supplementary Information:**

The online version contains peer-reviewed but unedited supplementary material available at 10.1007/s00125-025-06384-9.


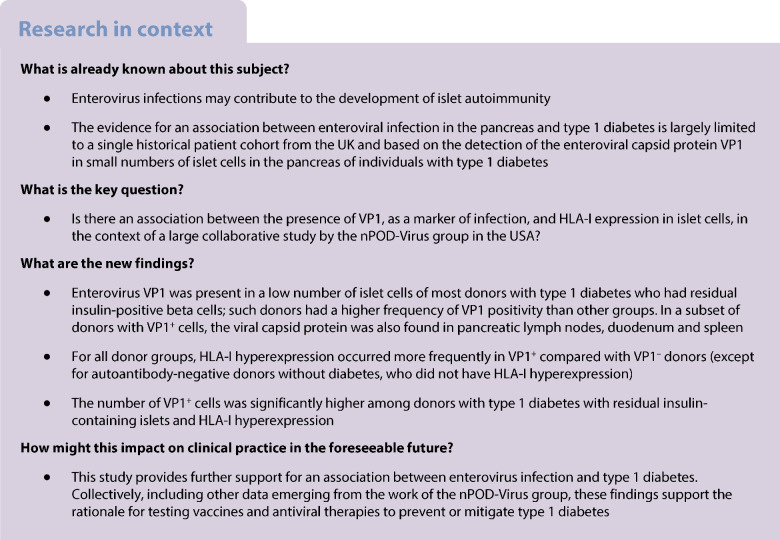


## Introduction

Substantial evidence supports an association of enteroviruses with type 1 diabetes [1–5]. Recently, the Environmental Determinants of Diabetes in the Young (TEDDY) study reported increased risk of islet autoimmunity in a genetically predisposed prospective cohort in the presence of coxsackievirus B (*Enterovirus B* species) in stool samples, especially when shedding was noted on multiple samples over time [3]; additional supporting evidence emerges from the prospective Finnish Type 1 Diabetes Prediction and Prevention study (DIPP) [6–9]. Pancreatic localisation of enterovirus infections and association with type 1 diabetes are supported by the more frequent detection of the virus capsid protein 1 (VP1) in islet cells of individuals with type 1 diabetes [10–13].

A hallmark feature of pancreas pathology in type 1 diabetes is the hyperexpression of HLA class I (HLA-I) molecules by islet cells, typically observed in insulin-containing islets (ICIs) [14–16]. Islet HLA-I hyperexpression has been observed in organ donors with diabetes-associated autoantibodies, considered in earlier stages of disease progression [17–19]. HLA-I hyperexpression is driven by IFNs, which can be induced by viral infections [20–22]. Whether hyperexpression of HLA-I in islet cells is associated with enteroviral infections as measured by VP1 positivity has not been determined.

Since inception in 2007, the Network for Pancreatic Organ Donors with Diabetes (nPOD) has obtained pancreas and other tissues from organ donors with type 1 diabetes and donors without diabetes expressing autoantibodies, providing impetus for a collaborative effort to investigate viral infections using synergistic approaches. In this broader context, this investigation focused on examining VP1 immunopositivity in the pancreas and other disease-relevant tissues (spleen, duodenum and pancreatic lymph nodes [PLNs]) and a potential association between VP1 positivity and HLA-I hyperexpression in islet cells.

## Methods

### Tissues from organ donors

**Fig. 1:**
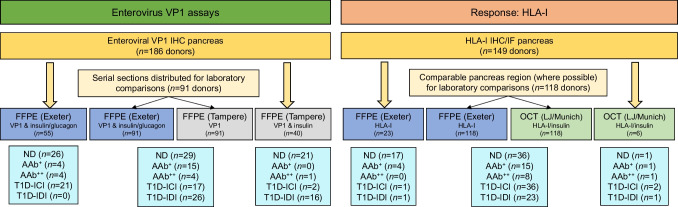
Distribution of pancreas samples, summary of the types of assays performed and the number of donors assessed/assay or assay combination. FFPE sections were provided to laboratories in Exeter (UK) and Tampere (Finland) and frozen sections to La Jolla (USA) and Munich (Germany). For both enterovirus VP1 and HLA-I assays, serial sections (enterovirus VP1) or sections from comparable pancreas regions (HLA-I) from a subset of donors were sent to Exeter/Tampere laboratories or to Exeter and La Jolla/Munich laboratories, respectively. All spleen, PLN and duodenum FFPE sections were distributed to Tampere and a subset of spleen samples (*n*=18) were sent to Exeter (ESM Tables 1 and 2). IF, immunofluorescence; IHC, immunohistochemistry; LJ, La Jolla; OCT, optimal cutting temperature compound

We studied 188 organ donors whose samples were provided by nPOD. Table 1 shows donor demographic information for each group, with individual donor data listed (including Research Resource Identifiers [RRiD]) in electronic supplementary material (ESM) Table 1. Eighty-three donors had type 1 diabetes, classified as having residual insulin-containing islets (T1D-ICI group, *n*=41) or only insulin-deficient islets (T1D-IDI group, *n*=42); we included donors without diabetes expressing a single autoantibody (AAb^+^ group, *n*=20) or multiple autoantibodies (AAb^++^ group, *n*=9), and autoantibody-negative donors without diabetes (non-diabetic [ND] group, *n*=76). We obtained consecutive 5 µm formalin-fixed paraffin-embedded (FFPE) and frozen sections from different regions of the pancreas (head, body and/or tail). We also studied FFPE sections from spleen and duodenum from 96 (37 ND, nine AAb^+^, four AAb^++^, 16 T1D-ICI and 30 T1D-IDI) and 70 (21 ND, nine AAb^+^, four AAb^++^, ten T1D-ICI and 26 T1D-IDI) donors, respectively (ESM Table 2). PLNs were available from ten donors (one AAb^++^; four T1D-ICI, five T1D-IDI). The majority of the donors were of white ethnicity, and we attempted to match control donors to donors with type 1 diabetes (58 autoantibody-negative donors without diabetes vs 66 donors with type 1 diabetes), as much as possible. We included all available individuals regardless of gender, which was provided in the medical charts. Research use of samples was allowed in donor consents and followed ethical institutional guidelines. All samples were de-identified and obtained by nPOD through its partnership organ procurement organisations as approved by the University of Florida Institutional Review Board (IRB), after consent for organ donation and research was obtained from family members. Figure 1 illustrates the sample distribution scheme from the nPOD Pathology Core to the participating laboratories.

**Table 1:** Summarised donor demographics

Donor group	ND	AAb^+^	AAb^++^	T1D-ICIs	T1D-IDIs
No. of donors	76	20	9	41	42
Age, years	23.6 (15.4–38.8)	26.0 (18.8–41.4)	23.0 (22.0–38.7)	22.0 (14.1–26.8)	30.9 (20.6–41.6)
Age range, years	0.3–75.0	0.2–66.0	17.7–69.2	5–79	4.4–78
Sex, *n* male/*n* female (% male)	45/31 (59.2)	13/7 (65.0)	5/4 (55.5)	19/22 (46.3)	21/21 (50.0)
BMI, kg/m^2^	24.2 (20.5–29.2)	23.7 (19.8–27.4)	26.0 (22.4–29.0)	24.3 (21.1–26.8)	24.3 (22.5–26.9)
Diabetes duration, years	NA	NA	N/A	5.0 (1.5–8.5)	15.0 (8.0–30.8)
Diabetes duration range, years	NA	NA	NA	0.0–56.0	1.5–74.0
C-peptide, nmol/l	1.3 (0.8–2.6)	1.2 (0.5–2.7)	1.8 (0.5–3.9)		
Donors with detectable C-peptide, *n* (%)				21 (51.2)	2 (4.8)

Data are shown as median (IQR) unless stated otherwise

### Immunohistochemistry and immunofluorescence

We examined pancreas sections from 188 donors, and sections were available for more than one pancreatic region from 70 donors (31 ND, nine AAb^+^, five AAb^++^, 15 T1D-ICI and ten T1D-IDI). The total number of sections per region analysed was: head *n*=62; body *n*=74; and tail *n*=84. Sections were stained for insulin, glucagon, VP1 and HLA-I. Staining for VP1 was performed using the anti-enterovirus VP1 clone 5D8/1 (Agilent, Stockport, UK). Its specificity has been demonstrated previously [23] and we further validated it by staining uninfected and virally-infected cell lines, and normal human tissue microarrays (TMA) (ESM Fig. 1). In Exeter, serial FFPE sections were heated in 10 mmol/l citrate (pH 6.0) in a microwave pressure cooker for 20 min, then cooled at room temperature for 20 min. The anti-VP1 monoclonal antibody (55 ng/ml) or the HLA-I (EMR8-5) monoclonal antibody (1/1500) (Abcam, Cambridge, UK) were incubated for 1 h at room temperature, and the EnVision HRP Detection System (Agilent) was used for antigen detection [10, 13, 23]. Serial sections were stained with anti-insulin antibody (catalogue no. A0564; Agilent; 1:600 for 1 h) and visualised using the Dako REAL Envision HRP detection system. Sections were subsequently stained with anti-glucagon antibody (K79bB10; Abcam, 1:2000 for 1 h) and visualised with the Vector AP-ABC kit combined with Vector Red substrate (Vector Laboratories, Newark, CA, USA). All slides were dehydrated and mounted in Agilent Fluorescence Mounting medium (Agilent). Sections were analysed by brightfield microscopy using either a Nikon 50i microscope fitted with a DS-Fi camera and DSL2 camera control unit, or the sections were scanned at 40× magnification using an Akoya Biosciences Vectra Polaris Automated Quantitative Pathology Imaging System. In Tampere, FFPE sections were stained with the same VP1 antibody (clone 5D8/1, sourced from DakoCytomation, Glostrup, Denmark; 1:300) using a Ventana BenchMark LT (Ventana Medical Systems) and the ultraView Universal detection systems, as described [12]. Consecutive pancreas sections were stained using an anti-insulin antibody (Ab-6; Thermo Scientific, 1:2000), as reported [24]. Sections were analysed by brightfield microscopy using either an Olympus BX60 microscope fitted with an Olympus Colorview III camera or scans of whole-slide images (SlideStrider scanner, Jilab, Tampere, Finland). In La Jolla/Munich, pancreatic frozen sections (*n*=118) were stained for insulin, glucagon and HLA-I. Tissue sections were fixed with 1% paraformaldehyde (% vol./vol.) and blocked with 2% goat serum (% vol./vol.). The following primary antibodies were incubated for 1 h at room temperature: polyclonal guinea pig anti-insulin (catalogue no. A0564, Agilent; 1:140), monoclonal recombinant rabbit anti-glucagon (catalogue no. ab92517, Abcam, UK; 1:400) and mouse monoclonal anti-human HLA-ABC (catalogue no. R7000, W6/32 clone; Agilent, 1:100). After incubation for 1 h at room temperature, and washes, sections were incubated with the following secondary, fluorescently labelled, antibodies: goat anti-guinea pig IgG (H+L)–Alexa Fluor 488; F(ab')2-goat anti-rabbit IgG (H+L)–Alexa Fluor 555; and goat anti-mouse IgG2a–Alexa Fluor 647 (1:1000; all from Invitrogen, Waltham, USA). Sections were counterstained with Hoechst 33342 (catalogue no. H3570, Invitrogen; 1:5000) for 8 min and mounted with Prolong Gold Antifade (catalogue no. P36930, Invitrogen). Sections were analysed manually using a Nikon digital DXM1200C camera and Nikon ACT-1C Camera Controller Software or scanned by an Axio Scan.Z1 slide scanner (Zeiss, Jena, Germany) using a 20×/0.8 numerical aperture (NA) Plan-Apochromat (a=0.55 mm) objective lens. Scanned sections were visualised using ZEN Blue 2.3 software (Zeiss, Jena, Germany). The analysis was performed by researchers blinded to the study groups.

### Criteria for enterovirus and HLA-I positivity

**Fig. 2:**
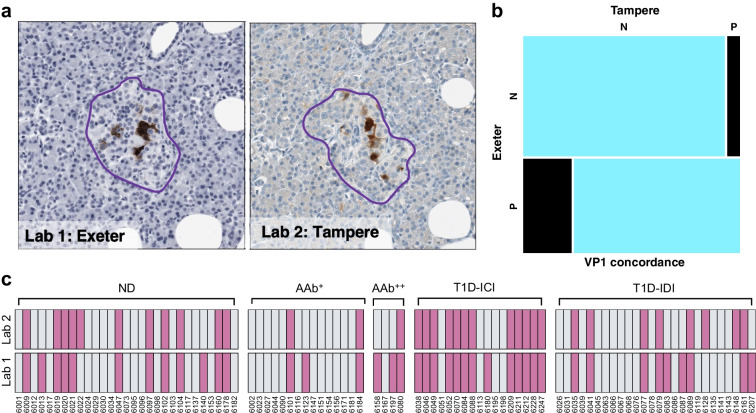
(**a**) Representative images of enterovirus VP1 staining in serial sections of an islet from nPOD donor 6084. The purple outline defines the islet. (**b**) Agreement of enterovirus VP1 immunostaining between laboratories. Samples taken from the same pancreatic block were evaluated in 91 donors in Exeter (Lab 1) and Tampere (Lab 2). Negative (N) or positive (P) agreement are coloured electric blue, and discordant results are displayed in black. A total of 48 samples were found to be negative for VP1 in both laboratories while 31 were positive. Three were negative in Exeter and positive in Tampere, whereas nine were positive in Exeter and negative in Tampere. (**c**) Comparison of blinded VP1 immunohistochemistry in two different laboratories (Lab 1, Exeter; Lab 2, Tampere) performed on serial sections. nPOD donor numbers are shown along the bottom. Grey, VP1^−^; pink, VP1^+^

For each donor and section, we evaluated the VP1 staining pattern in individual cells and classified them as VP1 negative (VP1^−^) or VP1 positive (VP1^+^), as shown in Fig. 2a. A donor was considered VP1^+^ in the presence of ≥1 strongly stained VP1^+^ cells within any islet of a section. If multiple sections or pancreatic regions were analysed per donor, a VP1^+^ cell in any of the sections/regions, from either of the different laboratories, was sufficient to define the donor as VP1^+^. Thus, any donor identified as VP1^+^ in either laboratory was recorded as VP1^+^, and any donor scored positive but analysed in a single laboratory was included. We classified islets into three categories according to their HLA-I staining intensity (normal, elevated and hyperexpression), as previously described [15, 19], if they had at least one islet in these categories.

### Quantification of VP1^+^ islets and cells

We quantified islets containing VP1^+^ cells. First, we determined the total number of islets per section using a serial section stained for insulin and glucagon. This was performed through manual counting or using the tissue classifier module of HALO V3.0 image analysis software (Indica Labs, Albuquerque, USA), where islets were defined as groups of endocrine cells covering an area of ≥1000 µm^2^ and/or comprising more than about ten insulin and/or glucagon positive cells. Then, we performed manual counting of VP1^+^ islets on the serially stained sections and calculated the proportion of VP1^+^ islets among the total islets examined. The number of VP1^+^ cells per section, the percentage of VP1^+^ islet cells and the percentage of VP1^+^ acinar cells were enumerated using the DenseNet AI V2 and Cytonuclear modules within the Indica Labs HALO Image analysis platform (version 3.2.1851.354). The DenseNet AI V2 was used to identify islet and acinar areas, where islets were defined as groups of endocrine cells covering an area of ≥1000 µm^2^. As a subsequent step, we used the Cytonuclear module to identify VP1^+^ cells across the total section, specifically within the islets or acinar area. VP1^+^ cells were identified as areas of staining above the background level by applying optimised threshold values for intensity and cell size. We applied this pipeline to all corresponding tissue sections. These analyses were performed for 116 donors (ND *n*=39; AAb^+^ *n*=18; AAb^++^ *n*=8; T1D-ICI *n*=33; T1D-IDI *n*=18).

### Statistical methods

We evaluated the agreement of VP1 and HLA staining assays performed in different laboratories; Gwet’s AC1 and AC2, respectively, were used to generate estimates of inter-rater reliability [25]. The strength of the agreement was based on a modification to a scale originally proposed for the κ coefficient [26]: 0.0–0.2 poor; >0.2–0.4 slight agreement; >0.4–0.6 moderate; >0.6–0.8 good; and >0.8–1.0 excellent. Negative and positive percentage agreement, including 95% CIs, are reported for each comparison. A weighted overall percentage agreement was computed based on the number of samples in each assessment. Two-sided Fisher’s exact tests with false discovery rate (FDR) corrections for multiple comparisons were used to determine differences in positivity between donor groups, Wilcoxon matched-pairs test when comparing islet and acinar compartments, and Kruskal–Wallis test to determine proportion of VP1^+^ cells in relation to HLA-I expression category. We examined the relationship between HLA and VP1 staining using univariate logistic regression (ULR) model testing. χ^2^ *p* value, the corresponding OR and the 95% CIs are reported. The profile likelihood method was used to generate the reported statistical measures. All statistical analysis was performed using SAS software, version 9.4 TS Level 1 M3 (SAS Institute, Cary, USA.), the R programming language version 3.6.1 (https://www.R-project.org/), or GraphPad Prism Version 10.2.3 (https://www.graphpad.com/). An SAS macro written by J. S. Uebersax was modified to calculate positive and negative agreement as well as 95% CIs (http://www.john-uebersax.com/stat/sp_sas.txt; last accessed 4 April 2021). The R package irrCAC [27], was used to calculate Gwet’s AC1 and AC2 agreement coefficient statistics. All *p* values are two-sided, unadjusted and were considered significant if α<0.05, unless otherwise indicated.

## Results

### VP1 positivity is associated with type 1 diabetes in the largest cohort of nPOD donors evaluated to date

We examined enterovirus VP1 in pancreatic islets from 186 nPOD donors, of whom 82 had type 1 diabetes. This is the largest cohort examined to date and it includes donors spanning a broad range of age and disease duration; 29 donors (35%) had disease duration <5 years (ESM Table 1). Importantly, 91 donors from all groups (including 43 donors with type 1 diabetes) were studied in two independent laboratories, using serial FFPE pancreas sections (Fig. 1). Each laboratory scored the VP1 immunopositivity in a blinded manner. Overall, there was 87% agreement between the Exeter and Tampere laboratories, resulting in good to excellent concordance of the VP1 datasets (*p*<0.001, Gwet’s AC1=0.75 [95% CI 0.61, 0.88]). Specifically, there was 89% negative (95% CI 83%, 95%) and 84% positive agreement (95% CI 74%, 93%) of all samples evaluated for VP1 by both laboratories (Fig. 2b). The inter-laboratory analysis of VP1 positivity for all the individual donors is illustrated as a heatmap in Fig. 2c.

**Fig. 3:**
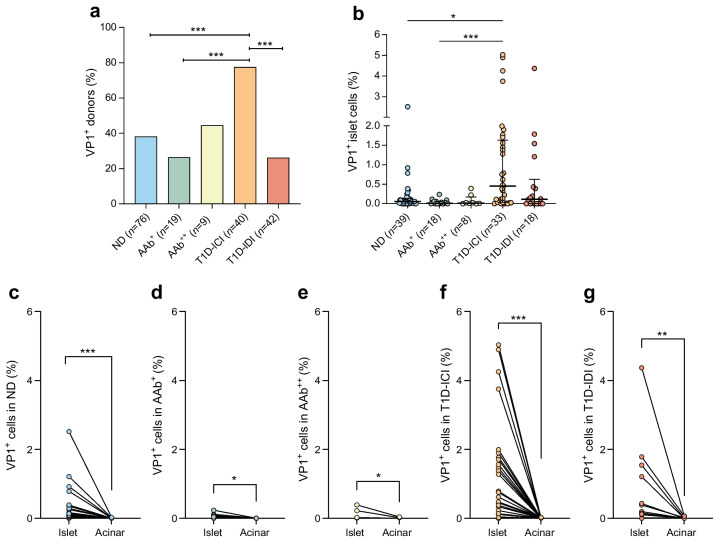
(**a**) Enterovirus VP1^+^ islet cells are more frequently observed in T1D-ICI donors. The bar graph shows significantly higher VP1 immunopositivity in the pancreas of T1D-ICI donors compared with ND, AAb^+^ and T1D-IDI donors (***p<0.001, two-sided Fishers Exact Test; significant after FDR corrections for multiple comparisons). (**b**) The frequency of enterovirus VP1^+^ islet cells is increased in donors with type 1 diabetes and residual beta cells (T1D-ICI). AI-based image analysis was performed on a subset of donor pancreas sections stained in Exeter to determine the proportion of islet cells that were VP1^+^ within each pancreatic section. Each data point represents an individual donor. The median, first and third quartile for the percentage of VP1^+^ islet cells are shown. *p<0.05, ***p<0.001 (one-way ANOVA with Kruskal–Wallis post-test). (**c**–**g**) Enterovirus VP1 is found more frequently in islet cells than in acinar cells in all donor groups. AI-based image analysis was performed on a subset of donor pancreas sections stained in Exeter to determine the proportion of islet or acinar cells that were VP1^+^. Each data point represents an individual donor with a line linking the proportion of islet and acinar VP1^+^ cells. Blue (**c**), ND (*n*=39); green (**d**), AAb^+^ (*n*=18); yellow (**e**), AAb^++^ (*n*=8); orange (**f**), T1D-ICI (*n*=33); red (**g**), T1D-IDI (*n*=18). *p<0.05, **p<0.01, ***p<0.001 (Wilcoxon matched-pairs test)

Based on this concordance, data were collated and analysed with the criteria described in Methods. Donors from four groups had an overall similar prevalence of VP1 positivity: 29/76 (38.2%) AAb^−^ donors without diabetes (ND group); 11/42 (26.2%) T1D-IDI donors; and among AAb-positive donors, 5/19 (26.3%) AAb^+^ and 4/9 AAb^++^ (44.4%) (Fig. 3a). In contrast, VP1 positivity was found at higher frequency among T1D-ICI donors (77.5%, 31/40, *p*<0.001 vs all groups except AAb^++^).

As previously reported [10, 13, 19], VP1^+^ islet cells are rare. We found a median of 0.054% VP1^+^ islet cells per tissue section in the ND group (*n*=39), and similarly low frequencies of 0.013% in AAb^+^ (*n*=18), 0.017% in AAb^++^ (*n*=8) and 0.12% in T1D-IDI (*n*=18) groups (Fig. 3b). In contrast, T1D-ICI donors (*n*=33) had a higher median proportion of VP1^+^ islet cells (0.45%, *p*<0.035 vs ND, *p*<0.0003 vs AAb^+^). We identified islet and acinar cells using the HALO DenseNet AI classifier and quantified the number of VP1^+^ cells in each compartment. VP1^+^ cells were consistently more abundant in the islet compartment in all donor groups (Fig. 3c–g). We also evaluated VP1 immunopositivity in spleen, PLNs and duodenum from a subset of donors, based on sample availability, in 96, 10 and 71 donors, respectively (ESM Tables 2 and 3). VP1^+^ cells occurred in all tissue types and all donor groups in the absence of statistically significant differences between groups (ESM Fig. 2a–d).

### Islet cell HLA-I hyperexpression is more frequent in AAb^++^ and T1D-ICI donors

**Fig. 4:**
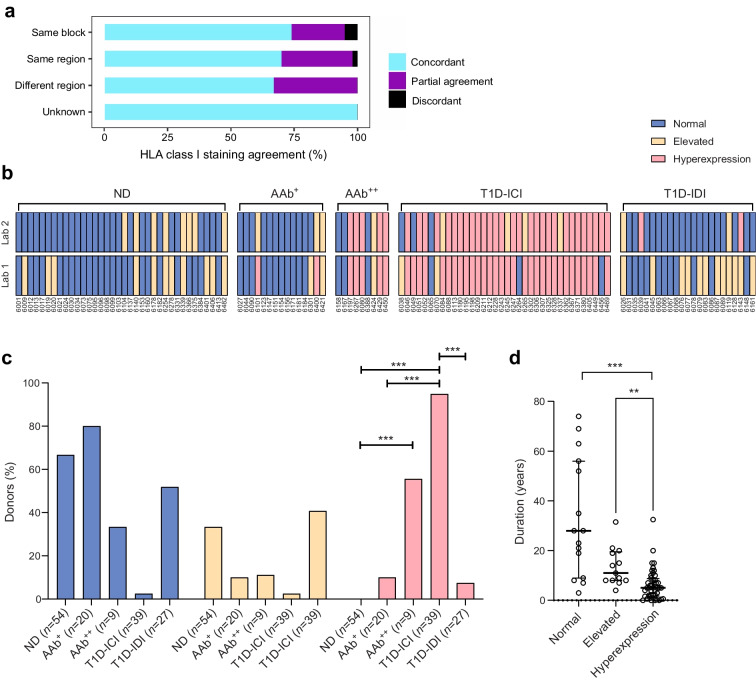
(**a**) Agreement of HLA staining by region of pancreatic sample. HLA staining from 118 donors was performed in Exeter and La Jolla/Munich laboratories using FFPE and optimal cutting temperature compound (OCT) frozen samples, respectively. Of those, samples were examined from the same block in 66 cases, a different block but the same region (i.e. head, body or tail) in 47 cases, different regions in three cases and unspecified blocks or regions in two cases. Normal expression, elevated expression or hyperexpression was noted by both laboratories. Complete agreement is coloured in electric blue, complete disagreement in black and partial agreement in violet. (**b**) Comparison of blinded HLA-I immunostaining in two different laboratories (Lab 1, Exeter; Lab 2, La Jolla/Munich) on FFPE vs OCT material respectively. Blue, normal expression of HLA-I; yellow, elevated expression of HLA-I; pink, hyperexpression of HLA-I. (**c**) HLA-I hyperexpression was more frequently observed in T1D-ICI and AAb^++^ donors compared with ND and T1D-IDI donors. Blue bars, proportion of donors with normal expression; yellow bars, elevated expression; pink bars, hyperexpression ***p<0.001 (two-sided Fishers Exact Test; two-sided significance after FDR corrections for multiple comparisons). (**d**) Donors with type 1 diabetes and HLA-I hyperexpression had overall shorter disease duration than those categorised as having elevated and normal expression. Each data point shows individual donor values for disease duration (in years) in each of the three HLA-I expression categories (normal, elevated and hyperexpression). The median, first and third quartile are shown. **p<0.01, ***p<0.001

We evaluated HLA-I expression in the islets of 149 donors, from all groups, among whom 118 were analysed in two independent laboratories. Samples were available from the following: (1) the same tissue block (66 cases); (2) a different tissue block but from the same region of the pancreas (i.e. head, body or tail) (47 cases); (3) tissue blocks from different regions of the pancreas (3 cases); and (4) tissue blocks from unspecified regions (2 cases) (Fig. 4a). Overall, there was good concordance between the results reported from each of the two laboratories (stratified weighted Gwet’s AC2=0.67). Good agreement (85%) between the two laboratories was obtained when samples were studied from the same tissue block (*p*<0.001, AC2=0.68, 95% CI 0.53, 0.83), or when different blocks from the same region were assessed (84% agreement; *p*<0.001, AC2=0.73, 95% CI 0.58, 0.88). Insufficient data were available to statistically evaluate the extent of concordance between laboratories when samples were drawn from either different or unknown regions of the pancreas. Figure 4b shows a heat map illustrating the agreement between the two laboratories. Based on the agreement achieved, data from both laboratories were collated and analysed using the following criteria: (1) an individual was reported as having HLA-I hyperexpression or elevated expression when it was noted in any islet by either laboratory; and (2) outcomes from donors analysed in a single laboratory were also included. In the ND group, 66.7% of donors had normal HLA-I expression, the remainder being classified as having elevated HLA-I expression; HLA-I hyperexpression was not detected (Fig. 4b, c). Conversely, HLA-I hyperexpression was found in most (37 of 39; 94.9%) T1D-ICI donors (*p*<0.001 vs ND); this was not restricted to donors with a recent diagnosis. HLA-I hyperexpression was also evident in T1D-ICI donors with longer disease duration (up to 32.5 years; median duration 5 years [IQR 1.7, 8.8]) and observed only in 7.4% (2 of 27) of T1D-IDI donors (*p*<0.001 vs T1D-ICI). Most T1D-IDI donors had normal HLA-I expression (14 of 27; 51.9%) vs 2.6% (1 of 39) of T1D-ICI donors (*p*<0.001). Across all donors with type 1 diabetes, those with HLA-I hyperexpression had overall shorter disease duration than those categorised as having elevated (*p*<0.01) and normal (*p*<0.001) HLA-I expression (Fig. 4d). In the autoantibody-positive group, HLA-I hyperexpression was noted in 10% (2 of 20) of AAb^+^ (*p*=0.07 vs ND) and 55.6% (5 of 9) of AAb^++^ individuals (*p*<0.001 vs ND) while 80% (16 of 20) and 33.3% (3 of 9) were classified as normal, respectively (Fig. 4c).

### Islet cell VP1 immunopositivity is associated with HLA-I hyperexpression in T1D-ICI donors

**Fig. 5:**
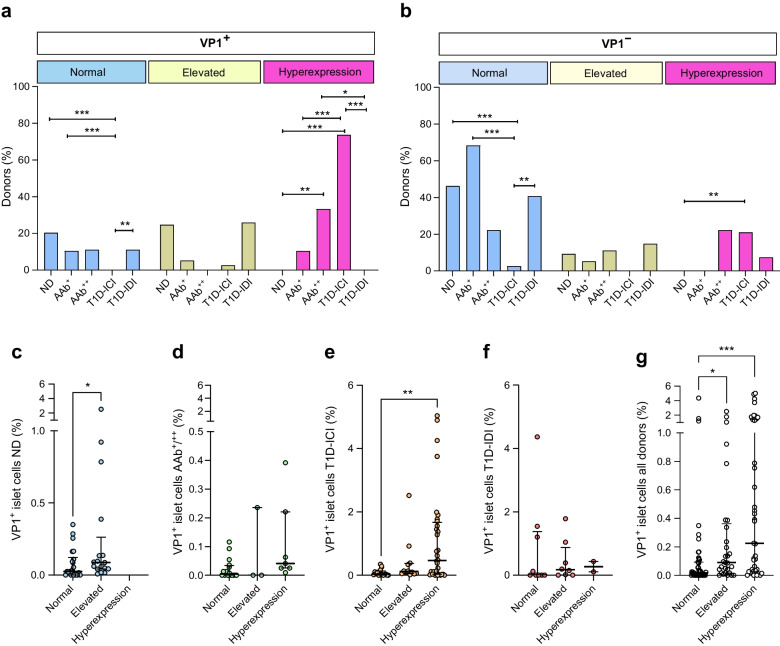
(**a**) Among VP1^+^ donors, HLA-I hyperexpression is more frequently observed in T1D-ICI and double AAb^++^ donors compared with ND donors. Bar graphs show the proportion of donors that are VP1^+^ and have normal expression (blue), elevated expression (yellow) or hyperexpression of HLA-I (pink) (*N*=72; ND *n*=24; AAb^+^ *n*=5; AAb^++^ *n*=4; T1D-ICI *n*=29; T1D-IDI *n*=10). *p<0.05, **p<0.001, ***p<0.001 (Kruskal–Wallis test). (**b**) HLA-I expression is more frequently normal in VP1^−^ ND, AAb^+^ and T1D-IDI donors (*N*=75; ND *n*=30; AAb^+^ *n*=14; AAb^++^ *n*=5; T1D-ICI *n*=9; T1D-IDI *n*=17). **p<0.01, ***p<0.001; two-sided Fishers Exact Test; two-sided significance after FDR corrections for multiple comparisons). (**c**–**g**) Dot plots show the proportion of VP1^+^ islet cells in relation to HLA-I expression category in each donor group and for all donors combined. The median, first and third quartile are shown. Blue (**c**), ND; green (**d**), AAb^+^/AAb^++^; orange (**e**), T1D-ICI; red (**f**) T1D-IDI; (**g**) all donors. *p<0.05, **p<0.001, ***p<0.001 (Kruskal–Wallis test)

We investigated whether an association exists between enterovirus VP1 positivity and HLA-I hyperexpression in islet cells. The various patterns of HLA-I and VP1 expression can be seen in ESM Fig. 3. VP1 and HLA-I immunostaining outcomes were available from 147 donors (54 ND, 19 AAb^+^, 9 AAb^++^, 38 T1D-ICI and 27 T1D-IDI). None of the pancreas samples from ND and T1D-IDI donors contained islets that were VP1^+^ and hyperexpressed HLA-I; in contrast, most T1D-ICI donors had both VP1 immunopositivity and HLA-hyperexpression (28/38, 73.7%, *p*<0.001 vs ND) (Fig. 5a). Some donors with islet autoimmunity also scored positive for both (10.5%, 2 of 19 of AAb^+^, *p*=0.065 vs ND; and 33.3%, 3 of 9 of AAb^++^, *p*=0.0021 vs ND). In those with normal HLA-I expression, VP1 immunopositivity was noted in a minority of ND (20.4%; 11 of 54), AAb^+^ (10.5%; 2 of 19), AAb^++^ (11.1%; 1 of 9) and T1D-IDI (11.1%; 3 of 27) donors, but never in T1D-ICI donors (*p*<0.001 vs ND; Fig. 5a). For all donor groups combined, HLA-I hyperexpression occurred more frequently in VP1^+^ than in VP1^−^ donors (45.8%, 33/72 vs 16%, 12/75, *p*<0.001, χ^2^ test).

We quantified VP1^+^ islet cells in relation to the HLA-I expression category in each donor group and for all donors combined (Fig. 5c–g). As noted, ND donors were not found to have islets with HLA-I hyperexpression, regardless of VP1 positivity; ND donors with elevated HLA-I status had a higher median frequency of VP1^+^ islet cells (*p*<0.05 vs normal HLA-I). Among T1D-ICI donors, the median frequency of VP1^+^ islet cells was higher in those with HLA-I hyperexpression than in those with normal expression (*p*<0.01). Examining all donors together, the frequency of VP1^+^ islet cells was highest in donors with HLA-I hyperexpression (*p*<0.001 vs normal HLA-I), intermediate in those with elevated expression (*p*<0.05 vs normal HLA-I) and lowest in those with normal expression (Fig. 5g).

In ULR modelling, relative to pancreatic samples with normal or elevated HLA-I, detection of VP1 positivity was 10.63 times (95% CI 1.66, 97.32; *p*=0.0186) more likely if hyperexpression was found among AAb^+^ or AAb^++^ donors but not the other donor groups (Table 2).

**Table 2:** ULR analysis

Donor group	VP1^−^		VP1^+^		OR	95% CI	*p* value
	*n*	Percentage	*n*	Percentage			
ND							
Normal to elevated HLA-I expression	30	100	24	100	–	–	–
HLA-I hyperexpression	0	0	0	0	–	–	–
AAb (AAb^+^ and AAb^++^)							
Normal to elevated HLA-I expression	17	89	4	44	–	–	–
HLA-I hyperexpression	2	11	5	56	10.63	1.66, 97.32	0.0186
T1D-ICI							
Normal to elevated HLA-I expression	1	11	1	3	–	–	–
HLA-I hyperexpression	8	89	28	97	3.50	0.13, 95.42	0.3941
T1D-IDI							
Normal to elevated HLA-I expression	15	88	10	100	–	–	–
HLA-I hyperexpression	2	12	0	0	0.295	0.002, 4.131	0.5295

Data are relative to pancreatic samples with normal or elevated HLA-I. Detection of VP1 positivity is 10.63 times more likely if hyperexpression is found; this relationship was present only in AAb-positive individuals (AAb^+^ and AAb^++^)

Finally, in a multivariate logistic regression model, we re-examined the relationship between HLA-I expression and VP1 immunopositivity in T1D-ICI donors according to disease duration. There was no collinearity between HLA-I expression and disease duration (Satterthwaite corrected *t* test *p*=0.3210) and the relationship between VP1 and HLA-I expression was independent of disease duration (*p*=0.4864). Specifically, simultaneously testing disease duration in a model with VP1 and HLA-I showed that for every additional year of type 1 diabetes, the likelihood of VP1 positivity was 0.97 (95% CI 0.897, 1.059) times less likely, but this was not significant (*p*=0.5697); moreover, relative to pancreatic samples with normal or elevated HLA-I, detection of VP1 was 1.39 times (95% CI 0.02, 51.78) more likely for every additional year of diabetes if hyperexpression was also present, but this was also not significant (*p*=0.8776). Thus, disease duration did not impact the association between VP1 and HLA-I hyperexpression.

## Discussion

Multiple studies have implicated enterovirus infections in type 1 diabetes [1–4]. Enteroviruses can impair beta cell function [28], promote inflammation and may trigger islet autoimmunity [3, 29, 30]. Earlier examinations of pancreas samples revealed the enteroviral capsid protein VP1 in islet cells from diabetic children and adults from geographically diverse cohorts; these studies involved pancreases obtained at autopsy from near diagnosis (Exeter Archival Diabetes Biobank [EADB] collection, UK, *n*=72) [10], from a subset (*n*=17) of nPOD donors with type 1 diabetes [13], and via biopsy in six newly diagnosed adults [16]. Our study represents the most extensive examination of pancreases from donors with type 1 diabetes (*n*=83, including the 17 previously investigated), identified by nPOD in the USA. Unlike the EADB cohort, consisting of autopsy cases identified between the 1940s and 1980s, this is a contemporary organ donor cohort collected between 2007 and 2019 across the USA; it also includes donors with broad age and disease duration ranges, including 29 donors with disease duration <5 years, vs only six in an earlier analysis of nPOD donors [13]. In addition, we examined VP1 positivity in the pancreas of the largest group to date (*n*=29) of rare organ donors expressing autoantibodies associated with type 1 diabetes, identified through the nPOD autoantibody screening of organ donors from the general population. Several pathological and functional alterations have been described in these donors [19, 31, 32], supporting the notion that this group represents a preclinical stage of type 1 diabetes. We included all available donors regardless of gender, and thus our approach observed gender equity. Given the number of donors examined in each group and that there is no clear sex bias reported for type 1 diabetes, gender was not formally included as a variable in our analysis.

This study is part of a comprehensive effort undertaken by the nPOD-Virus group. In this setting, we tested concordance and reproducibility by adopting a workshop format, and blinded samples were co-ordinately examined by multiple laboratories using multiple techniques [33, 34]. Here, we employed the monoclonal antibody clone 5D8/1 to detect enteroviral VP1 in tissue samples, since the specificity of this reagent had been extensively verified in earlier work [23] and in our unpublished data (Laiho JE, Zeissler M, Morgan NG, Hyöty H, Richardson SJ), and further validated here (ESM Fig. 1). Our analyses were conducted in a blinded fashion by two different laboratories. The extent of concordance of all findings (whether negative or positive) across laboratories was good (typically >85% between the two laboratories). This is remarkable given the very low prevalence of VP1^+^ islet cells.

Our results confirm, expand and rigorously validate earlier findings showing increased frequency of VP1 positivity in islet cells of donors with type 1 diabetes and residual beta cells (77.5% in our study), being significantly higher than in similarly aged donors without diabetes and the other groups considered (*p*<0.001, except vs AAb^++^ donors). Moreover, we quantified the abundance of VP1^+^ cells and report that T1D-ICI donors had a higher median proportion of VP1^+^ islet cells than any other group. Among T1D-ICI donors, islet cell VP1 immunopositivity was not restricted to donors with short disease duration, as it was also detected in those with longer duration (median 5 years, up to 34.5 years, provided that residual ICIs were present). A major determinant of VP1 positivity was indeed the presence of ICIs, further linking enteroviruses to beta cells and type 1 diabetes. Less than 25% of T1D-IDI donors had VP1^+^ cells in their insulin-negative islet cells. Our observations and previous literature [13] concur in suggesting that islet cells are the preferential target of enteroviral infections in the pancreas (Fig. 3c–g), with the percentage of VP1^+^ cells being higher in islets than in acinar cells. In a subset of donors with VP1^+^ islet cells, the viral capsid protein could also be detected in other organs, including spleen, duodenum and/or PLNs, in further support of an ongoing enterovirus infection. The primary replication of enteroviruses takes place in the respiratory tract and/or gut mucosa (including in the lymphoid tissues found at these locations), from where the virus can spread to internal organs such as pancreas, spleen, heart and the central nervous system via the systemic circulation. Thus, extra-pancreatic tissues may act as a reservoir from which the virus may reach the pancreas. Enteroviral particles could possibly be carried by immune cells, perhaps from islet to islet, noting that enterovirus RNA was detected in CD45^+^ cells near pancreatic islets in a previous study from our group [35]; enteroviral RNA is also present in blood cells from AAb-positive individuals, especially in antigen presenting cells [36]. Considering the low frequency of VP1^+^ cells in all groups, and the persistence of VP1 immunopositivity well beyond the time of diagnosis, these results suggest that islet enteroviral infections may be low grade or evolve into a low grade after an initial acute infection. An acute infection stage characterised by extensive cell lysis and the release of large numbers of viral particles was not observed in our study, yet it cannot be excluded. VP1^+^ islet cells might represent a reservoir of virus and reflect a relative inability to completely eradicate the infection. Related, 5′ terminally-deleted, replication-deficient enteroviruses forms have been reported in both heart and mouse pancreas, and are associated with pathology and virus persistence [37–39], which may promote chronic inflammation.

Enterovirus infections and detection of VP1^+^ islet cells are not exclusive to individuals with type 1 diabetes. While this can be considered to challenge a role for enteroviruses in type 1 diabetes, the discrepancy may actually reflect other factors (genetic, immunological, etc.) that may impact the outcome of the infection. To this end, we focused on the host response by examining hyperexpression of HLA-I by islet cells and its relationship to VP1 immunopositivity. Earlier studies suggested that upregulation of islet HLA-I molecules precedes the onset of clinical disease, as HLA-I hyperexpression can be detected in some AAb^++^ individuals [18, 19]. These donors would be reasonably considered to have reached, at minimum, the preclinical stage 1 of type 1 diabetes, as defined by the presence of multiple AAbs [40]. HLA-I hyperexpression is regarded as a hallmark feature of pancreas pathology in type 1 diabetes. While HLA-I hyperexpression may be determined by several inflammatory signals, it is strongly promoted by IFN-α, which is released by most cells (including beta cells) in response to viral infection. A strong association of IFN-α with islet cell HLA-I expression has already been shown in type 1 diabetes [16, 20, 21, 41, 42], and we focused on investigating the relationship between islet cell VP1 immunopositivity and HLA-I hyperexpression. Of note, only a single plane of each islet was studied in any given pancreas section; since VP1^+^ cells are rare, we cannot exclude that an islet scored as VP1^−^ might contain VP1^+^ cells outside the plane studied. With this caveat, we assessed the frequency at which VP1 immunopositivity occurs in the presence or absence of HLA-I hyperexpression, by donor groups. As we did with the VP1 analysis, assessment of HLA-I expression was conducted by two independent laboratories for 118 of 149 donors, with good concordance. Hyperexpression of HLA-I was seen in most T1D-ICI donors and was not limited to those with a recent diagnosis, as long as ICIs were retained. In contrast, among VP1^+^ donors, none of the pancreases from the ND and T1D-IDI groups contained islets that were VP1^+^ and hyperexpressed HLA-I. For all donor groups, HLA-I hyperexpression occurred more frequently in VP1^+^ donors than in VP1^−^ donors (*p*<0.001). These novel phenomenological associations, which we support with statistical significance, suggest the hypothesis that enterovirus infection can be a trigger for HLA-I hyperexpression, although we fully acknowledge that other stimuli may be involved as well, and that the analysis of tissues does not allow to infer causality. Of note, HLA-I hyperexpression was not seen in ND donors, even those with VP1 positivity; this suggests that how, and to what extent, beta cells respond to viral infections may be critical for disease risk and progression. Such differences in responses may be explained by the genetic background, noting that type 1 diabetes susceptibility genes (e.g. *IFIH1*) may control innate responses to infection in both beta cells and immune cells. Furthermore, analysis of disease risk genes expressed in beta cells using Ingenuity Pathway Analysis has revealed top hits in pathways associated with ‘Interferon signalling’, the ‘Role of JAK1, JAK2 and TYK in IFN signalling’ and the ‘Role of pattern recognition receptors in recognition of virus and bacteria’ [43], supporting a more pronounced response to infection in those at genetic risk for type 1 diabetes. Other possible scenarios include the underlying autoimmune/inflammatory process, which could make beta cells more susceptible to infections and simultaneously trigger HLA-I upregulation on islet cells. VP1 expression may also reflect a non-cytopathic infection that chronically stimulates the antiviral immune response, promoting the upregulation of HLA-I.

This study has several limitations. Due to the age distribution of organ donors, we studied relatively few children, in whom the evidence implicating enterovirus infection is strongest [10, 13]. Our study also examined tissues from rare islet AAb-positive organ donors, and in some cases we could find evidence of VP1^+^ and HLA-I hyperexpression in the islets.

In conclusion, we present the most extensive analysis to date of pancreas specimens from organ donors at various stages (preclinical and clinical) of type 1 diabetes in whom we examined the prevalence of VP1 positivity and HLA-I expression in islet cells. The strong concordance between independent laboratories supports the reproducibility of our findings, proving robust evidence that VP1 positivity is commonly detected in the pancreas of individuals with type 1 diabetes who retain residual insulin-positive beta cells; this links enteroviral infections to the islet cell population lost in type 1 diabetes. We further demonstrate a previously unknown association between VP1 positivity and HLA-I hyperexpression in islet cells. Moreover, higher proportions of VP1^+^ cells were found in islets with HLA-I hyperexpression, specifically in donors with type 1 diabetes and residual beta cells. The absence of extensive beta cell lysis and acute viral infection, even among donors examined near diagnosis or at the stage of multiple AAb positivity, and the presence of VP1^+^ cells in insulin-positive islets several years after diagnosis support the hypothesis of a low-grade, possibly persisting or recurrent enterovirus infection that could trigger IFN responses, leading to or contributing to the hyperexpression of HLA-I molecules. This is consistent with recent epidemiological studies [3]. Therefore, this mode of enteroviral infection in type 1 diabetes is now supported by epidemiological and pancreas pathology evidence.

## Supplementary Information

Below is the link to the electronic supplementary material.

ESM Table 1 (XLSX 28.1 KB)

ESM (PDF 1.98 MB)

## Data Availability

Data generated and analysed during this study are available through the corresponding author upon request.

## Abbreviations

AAb: Autoantibody
EADB: Exeter Archival Diabetes Biobank
FDR: False discovery rate
FFPE: Formalin-fixed paraffin-embedded
HLA-I: HLA class I
ICI: Insulin-containing islet
IDI: Insulin-deficient islet
ND: Non-diabetic
nPOD: Network for Pancreatic Organ Donors with Diabetes
PLN: Pancreatic lymph node
T1D-ICI: Type 1 diabetes with insulin-containing islets (group)
T1D-IDI: Type 1 diabetes with insulin-deficient islets (group)
ULR: Univariate logistic regression
VP1: Virus capsid protein 1
