# Supplementary material for: Enterovirus VP1 protein and HLA class I hyperexpression in pancreatic islet cells of organ donors with type 1 diabetes
Source: Diabetologia. 2025 Mar 17;68(6):1197–210. doi: 10.1007/s00125-025-06384-9 (PMC12069150; doi:10.1007/s00125-025-06384-9)
Supplement: Supplementary file 2 — ESM (PDF 1.98 MB) [file 125_2025_6384_MOESM2_ESM.pdf]

ESM Table 2: Distribution of donors and assays performed

	Pancreas		Spleen	Duodenum	PLN
**Donor Group**	**VP1 IHC**	**HLA Class I**	**VP1 IHC**	**VP1 IHC**	**VP1 IHC**
No Diabetes	76	54	37	21	0
Single AAb positive (AAb+)	19	20	9	9	0
Dual Aab positive (AAb++)	9	9	4	4	1
Type 1 diabetes Residual ICIs	40	39	16	10	4
Type 1 diabetes IDIs only	42	27	30	26	5
TOTAL	186	149	96	70	10

ESM Table 3: Distribution of donors and assays performed

**Donor Group**	Spleen		Duodenum		PLN	
	**Total (n)**	**No. VP1+ (%)**	**Total (n)**	**No. VP1+ (%)**	**Total (n)**	**No. VP1+ (%)**
No Diabetes	37	9 (24.3%)	21	3 (14.3%)	0	-
Single AAb positive (AAb+)	9	3 (33.3%)	9	4 (44.4%)	0	-
Dual Aab positive (AAb++)	4	1 (25%)	4	1 (25%)	1	1 (100%)
Type 1 diabetes Residual ICIs	16	8 (50.0%)	10	3 (30%)	4	2 (50%)
Type 1 diabetes IDIs only	30	13 (43.3%)	26	12 (46.2%)	5	3 (60.0%)
**All T1D**	46	21 (45.7%)	36	15 (41.7%)	9	5 (55.5%)
**AAb+, AAb++ and T1D**	59	25 (42.4%)	49	20 (40.8%)	1	1 (100%)
**TOTAL**	96	34 (35.4%)	71	23 (32.4%)	10	6 (60%)

N/S by Fishers Exact Test

ESM Figure 1

a.

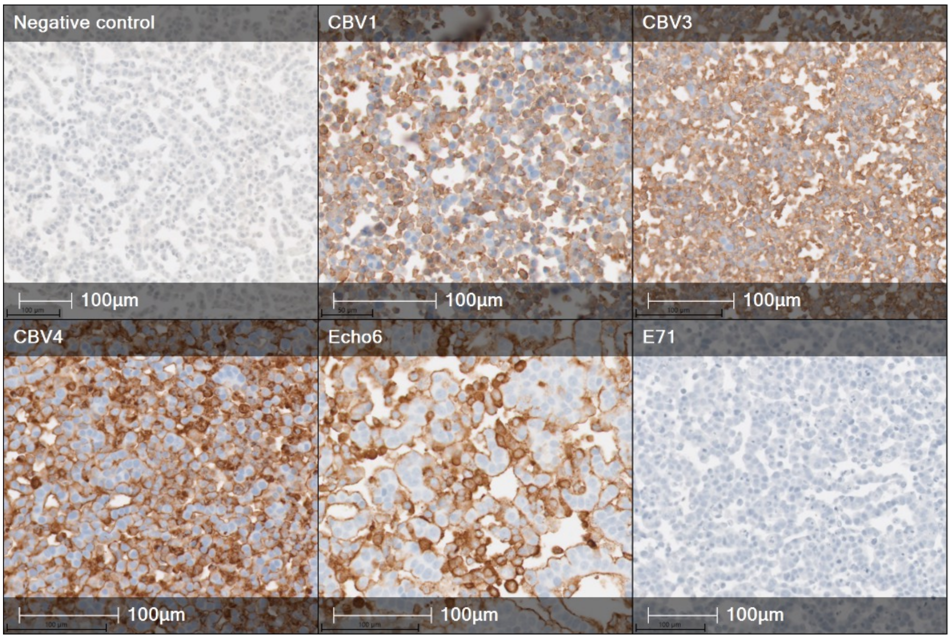

b.

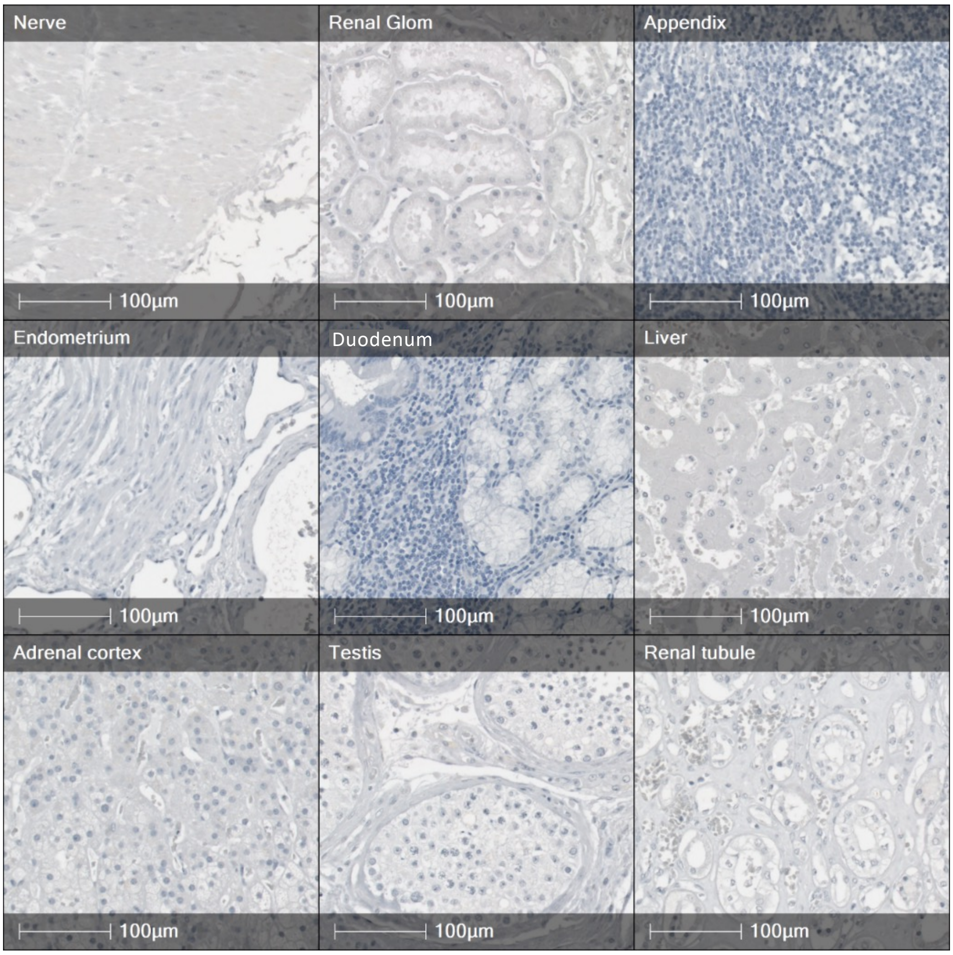

ESM Figure 1a: Enteroviral VP1 immunostaining in a virally-infected cell microarray. Representative immunostaining of VP1 in uninfected control cells (negative control), and cells infected with CVB1, CVB3, CVB4, echovirus 6 (Echo6) and enterovirus 71 (E71). Positive staining was observed in CVB1, 3, 4 and echo6, while no staining was seen in uninfected control cells or E71.

ESM Figure 1b: Enteroviral VP1 immunostaining in normal tissue microarray. Nerve, renal glomeruli, appendix, endometrium, duodenum, liver, adrenal cortex, testis and renal tubules were all negative for VP1. Scale bars - 100µm.

ESM Figure 2

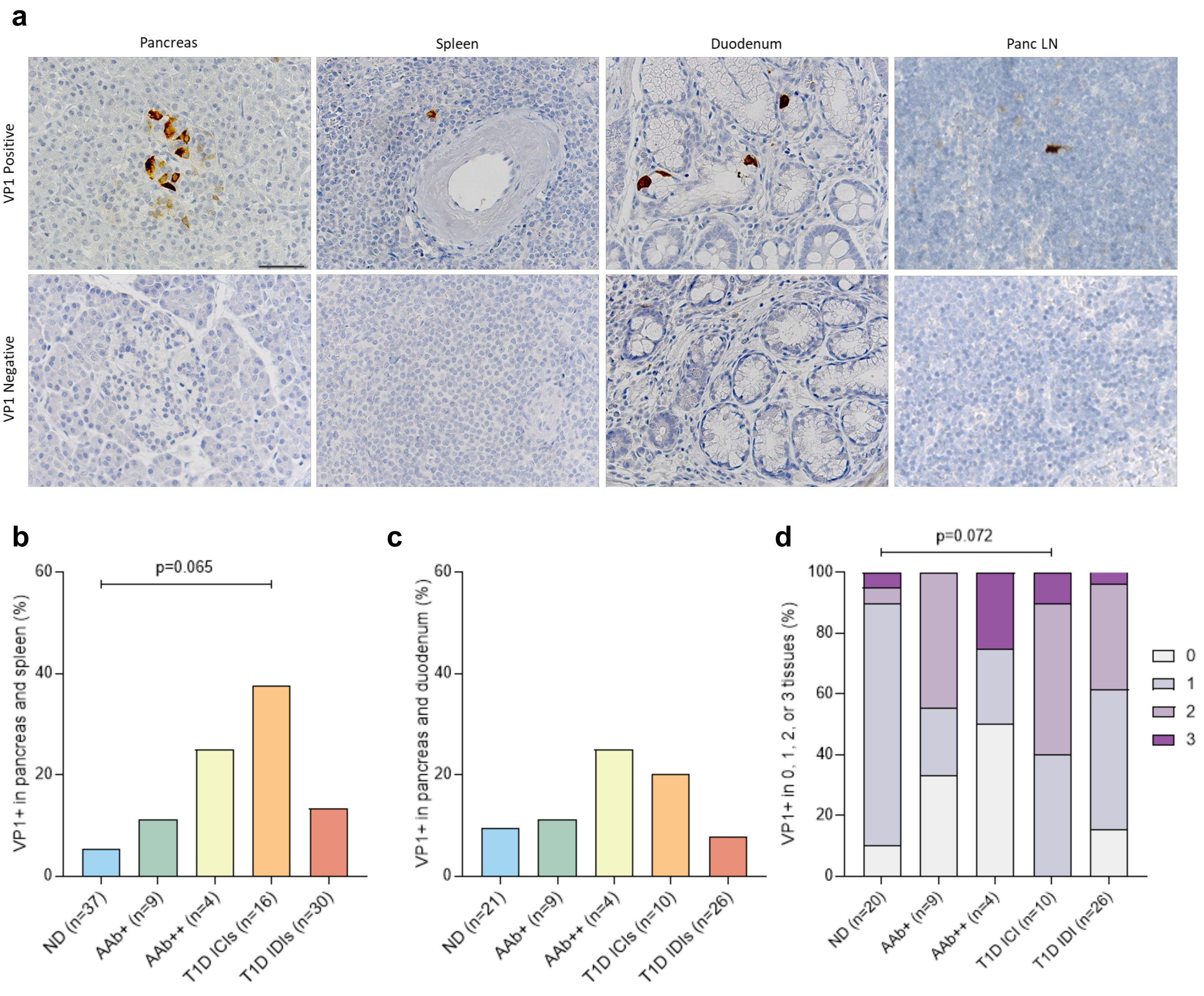

ESM Figure 2a. Representative images of VP1+ cells in nPOD donor pancreas, spleen, duodenum and PLN, compared with VP1- donors.

ESM Figure 2b. The proportion of donors in each donor group that have VP1 immunopositivity in both the spleen and pancreas.

ESM Figure 2c. The proportion of donors in each donor group that have VP1 immunopositivity in both the duodenum and pancreas.

ESM Figure 2d. The proportion of donors in each donor group that have VP1 immunopositivity in 0, 1, 2, or 3 different organs (pancreas, spleen and duodenum).

Two sided Fishers Exact Test with significance shown after FDR corrections for multiple comparisons.

ESM Figure 3

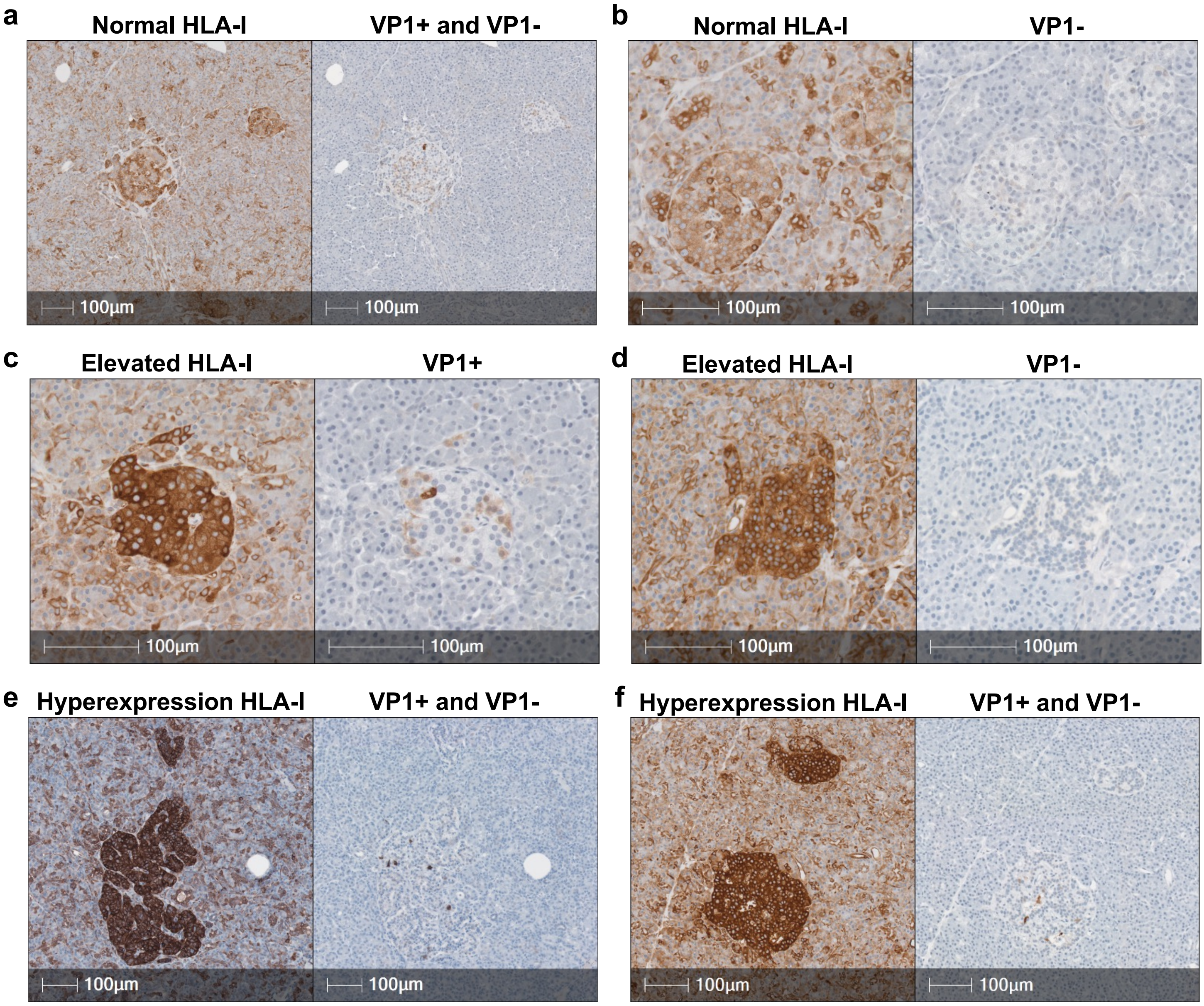

ESM Figure 3a. Representative images of two islets with normal HLA-I expression, one containing a VP1+ cell and one VP1-.

ESM Figure 3b. Representative images of two islets with normal HLA-I expression, both VP1-.

ESM Figure 3c. Representative images of one islet with elevated HLA-I expression containing a VP1+ cell.

ESM Figure 3d. Representative images of one islet with elevated HLA-I expression and VP1-.

ESM Figure 3e. Representative images of two islets with HLA-I hyperexpression, one containing several VP1+ cells and one VP1-.

ESM Figure 3f. Representative images of two islets with HLA-I hyperexpression, one containing several VP1+ cells and one VP1-.

nPOD-Virus group members:

nPOD-V Funding 2012: Funding Reference Breakthrough T1D (former JDRF) JDRF-25-2012-516

Participants:

- Pugliese, Alberto, M.D. Arthur Riggs Diabetes & Metabolism Research Institute, City of Hope, Duarte, CA, USA.
- Atkinson, Mark, Ph.D. University of Florida, USA.
- Campbell-Thompson, Martha University of Florida, USA.
- Chapman, Nora, Ph.D. University of Nebraska, USA.
- Coppieters, Ken University of Ghent, Belgium.
- Dotta, Francesco, M.D. University of Siena, Italy.
- Eisenbarth, George, M.D., Ph.D. Barbara Davis Center for Childhood Diabetes, USA.
- Ferreira, Ricardo, Ph.D JDRF/WT Diabetes & Inflammation Laboratory, USA.
- Frisk, Gun, Ph.D. University of Uppsala, Sweden.
- Gianani, Roberto, M.D. Barbara Davis Center for Childhood Diabetes, USA.
- Gerling, Ivan, Ph.D University of Tennessee, USA.
- Homann, Dirk, M.D. University of Colorado at Denver, USA.
- Hyöty, Heikki, Ph.D. Tampere University, Finland.
- Lloyd, Richard, Ph.D. Baylor College of Medicine, USA.
- Kaddis, John, Ph.D. City of Hope National Medical Center, USA.
- Kent, Sally, Ph.D. University of Massachusetts, USA.
- Morgan, Noel, Ph.D. University of Exeter, UK.
- Nadler, Jerry, M.D., Ph.D. East Virginia Medical School, USA.
- Morris Fear, Margareta, Ph.D. East Virginia Medical School, USA.
- Nyalwhide, Julius, Ph.D. East Virginia Medical School, USA.
- Oikarinen, Maarit Tampere University, Finland.
- Plagnol, Vincent, Ph.D. University College of London, UK.
- Petrosino, Joseph, Ph.D. Baylor College of Medicine, USA.
- Richardson, Sarah, Ph.D. University of Exeter, UK.
- Sarkar, Suparna Barbara Davis Center for Childhood Diabetes, USA.
- Schneider, Darius La Jolla Institute for Allergy & Immunology, USA.
- Thackray, Larissa, Ph.D. Washington University, USA.
- Toniolo, Antonio. M.D. University of Insubria, Italy.
- Virgin, Herbert Washington University, USA.
- Von Herrath, Matthias La Jolla Institute for Allergy & Immunology, USA.

nPOD-V Grant 2017: Funding Reference Breakthrough T1D (former JDRF) JDRF-3-SRA-2017-492-A-N

Participants:

- Alberto Pugliese, Arthur Riggs Diabetes & Metabolism Research Institute, City of Hope, Duarte, CA, USA.

- Richard Lloyd, Baylor College of Medicine, USA.
- Margaret Morris, Eastern Virginia Medical School, USA.
- Roberto Mallone, INSERM, Paris, France.
- Malin Flodström Tullberg, Karolinska Institutet, Sweden.
- Matthias von Herrath, La Jolla Institute for Allergy and Immunology, USA.
- Jerry Nadler, Eastern Virginia Medical School, USA.
- Julius Nyalwidhe, Eastern Virginia Medical School, USA.
- Teresa Rodriguez-Calvo, La Jolla Institute for Allergy & Immunology, USA and Helmholtz Munich, Germany.
- Sally Kent, University of Massachusetts Medical School, USA.
- Antonio Toniolo, University of Insubria, Italy.
- Kathrin Maedler, University of Bremen, Germany.
- Marc Horwitz, University of British Columbia, Canada.
- Noel Morgan, University of Exeter Medical School, UK.
- Sarah Richardson, University of Exeter Medical School, UK.
- Mark Atkinson, University of Florida, USA.
- Ivan Gerling, The University of Tennessee Health Science Center, USA.
- Heikki Hyöty, Tampere University, Finland.
- Isaac Snowhite, University of Miami School of Medicine, USA.
- Filippo Canducci, University of Insubria, Italy.
- Alessandro Salvatoni, University of Insubria, Italy.

Additional members (some participated in discussions, but their participation in the group was more informal).

- Soile Tuomela PhD (Karolinska Institutet, Sweden)
- Ben Giepmans PhD (University of Groningen, Netherlands)
- Varpu Marjomaki PhD (University of Jyväskylä, Finland)
